# Angiotensin II modulates THP-1-like macrophage phenotype and inflammatory signatures via angiotensin II type 1 receptor

**DOI:** 10.3389/fcvm.2023.1129704

**Published:** 2023-08-25

**Authors:** Tlili Barhoumi, Fatmah A. Mansour, Maroua Jalouli, Hassan S. Alamri, Rizwan Ali, Abdel Halim Harrath, Maha Aljumaa, Mohamed Boudjelal

**Affiliations:** ^1^ Medical Research Core Facility and Platforms (MRCFP), King Abdullah International Medical Research Center/King Saud bin Abdulaziz University for Health Sciences (KSAU-HS), King Abdulaziz Medical City (KAMC), NGHA, Riyadh, Saudi Arabia; ^2^ Department of Biology, College of Science, Imam Mohammad Ibn Saud Islamic University (IMSIU), Riyadh, Saudi Arabia; ^3^ Department of Clinical Laboratory Sciences, College of Applied Medical Sciences, King Saud bin Abdulaziz University for Health Sciences/King Abdullah International Medical Research Center, Riyadh, Saudi Arabia; ^4^ Department of Zoology, College of Science, King Saud University, Riyadh, Saudi Arabia; ^5^ Department of Biology, College of Science, Princess Nourah Bint Abdulrahman University, Riyadh, Saudi Arabia

**Keywords:** angiotensin II, macrophages, ROS, inflammation, angiotensin II type 1 receptor (AT1R)

## Abstract

Angiotensin II (Ang II) is a major component of the renin–angiotensin or renin–angiotensin–aldosterone system, which is the main element found to be involved in cardiopathology. Recently, long-term metabolomics studies have linked high levels of angiotensin plasma to inflammatory conditions such as coronary heart disease, obesity, and type 2 diabetes. Monocyte/macrophage cellular function and phenotype orchestrate the inflammatory response in various pathological conditions, most notably cardiometabolic disease. An activation of the Ang II system is usually associated with inflammation and cardiovascular disease; however, the direct effect on monocyte/macrophages has still not been well elucidated. Herein, we have evaluated the cellular effects of Ang II on THP-1-derived macrophages. Ang II stimulated the expression of markers involved in monocyte/macrophage cell differentiation (e.g., CD116), as well as adhesion, cell–cell interaction, chemotaxis, and phagocytosis (CD15, CD44, CD33, and CD49F). Yet, Ang II increased the expression of proinflammatory markers (HLA-DR, TNF-α, CD64, CD11c, and CD38) and decreased CD206 (mannose receptor), an M2 marker. Moreover, Ang II induced cytosolic calcium overload, increased reactive oxygen species, and arrested cells in the G1 phase. Most of these effects were induced via the angiotensin II type 1 receptor (AT1R). Collectively, our results provide new evidence in support of the effect of Ang II in inflammation associated with cardiometabolic diseases.

## Introduction

Angiotensin II (Ang II) is the major component of the renin–angiotensin system (RAS), which is responsible for the regulation of vascular resistance and systemic blood volume. Ang II is produced from angiotensin I and angiotensin-converting enzyme (ACE) (1) from the liver and kidneys and after renin intervention at the upper level (2). Also, Ang II may be found in several compartments of the human host, which usually interact with the regulatory system in parallel (3). Ang II blockers are widely used as a preventive measure against a variety of pathological conditions, most notably cardiometabolic disease. Ang-II-induced hypertension and end-organ damage are usually accompanied by oxidative stress, fibrosis, cell apoptosis, and inflammation (4). Studies have generally reported a proinflammatory profile and activation of immune responses when RAS is highly activated (5). We and others recently reported that Ang II plays a major role in innate and adaptive immunity associated with hypertension and vascular injuries (6–8). In addition, Ang-II-induced organ damage is associated with dysfunction of the myelomonocytic cells, as well as massive infiltration of monocyte/macrophage into the tissue of vital organs, such as the brain, heart, kidneys, and aorta (9).

Monocytes/macrophages express various RAS elements, such as ACE1/2 and Ang II receptors (ATRs). The expression of ACE and ATRs in monocyte/macrophage highlights their local or systemic role as a regulator of low-grade inflammation and immune functions; thus, dysfunction of RAS components may promote vascular damage and endothelial dysfunction in hypertension (10). Monocyte/macrophage cells are characterized by their plasticity to change phenotype throughout differentiation and polarization dependent on physiologic or pathophysiologic microenvironment conditions and intra- and extracellular stimuli. Thus, the final status of monocyte/macrophage cells is still not completely understood. We aimed to study the effect of Ang II on THP-1-like macrophage phenotype and inflammatory signatures.

## Materials and methods

### Cell culture

THP-1 cell line (TIB-202) was purchased from ATCC and cultured in Roswell Park Memorial Institute (RPMI) 1640, supplemented with 2-mercaptoethanol (0.05 mM), 10% fetal bovine serum (Gibco), and 1% penicillin/streptomycin antibiotic (Gibco), and incubated in 37°C at 5% CO_2_ in humidified incubator. To differentiate cells, we stimulate them with 100 nM phorbol-12-myristate 13-acetate (PMA) (Millipore/Sigma) and ionomycin (1 µg/ml; Sigma) for 3 days. The PMA-supplemented media was removed by washing two times with phosphate-buffered saline (PBS) and incubated with fresh PMA-free media, then treated with Ang II (1 µM) for another 24 h. We start our experiments with 100 nM and 1 µM, but consistent results for all markers were not found. We chose 1 µM to boost to maximum the effect of Ang II that may be hidden for some markers. To address for relevance to human cells, additional studies were performed using human-derived macrophages. The monocytes isolated by plastic adherence from human PBMCs after incubation at 37° for 3 h in RPMI 1640 were stimulated for 5 days with rhM-CSF (50 ng/ml) (Thermo Fisher Scientific) to complete the macrophage differentiation. For M1 polarization, the cells were further incubated for 24 h with 100 ng/ml of LPS and 20 ng/ml of human IFN-γ.

### Analysis of cell death and apoptosis

To evaluate the cytotoxicity of Ang II, the cells were resuspended in 400 µl of PBS, stained with propidium iodide (PI) (10 µg/ml) for 15 min at room temperature, and immediately analyzed by flow cytometer (BD FACSCanto II). An immunofluorescence imaging using the EVOS® FL Auto Imaging System (Thermo Fisher Scientific) was performed to confirm the flow cytometry experiment. The PI/Hoechst 33342 (10 µg/ml) combination staining was used to assess cell necrosis and apoptosis. After 15 min incubation with Hoechst at 37°C, the cells were centrifuged, and the pellets were resuspended in 300 µl PI (10 µg/ml) staining solution at room temperature for another 15 min. Immediately, the cells were analyzed by flow cytometry using (470/50 nm) emission light for Hoechst and (570/30 nm) for PI detection.

### Measurement of cell phenotype changing and THP1-like macrophage polarization

Some of the cells were treated with 1 µM Ang II for 24 h, and other cells without Ang II treatment. After washing with PBS, the cells were resuspended in FACS buffer (100 µl), then stained with corresponding conjugated antibodies at 1 µg/ml for the following markers: PE-conjugated mAb specific for CD15, CD33, and CD44; FITC-conjugated mAb specific for CD49F, CD64, and CD116; and Percp-conjugated mAb specific for CD38. The PE-conjugated mAb for TNF-*α* and CD11C were used as M1 markers in a separate experiment, while to identify M2 cells, CD206 was used as the specific antibody. After incubation for 30 min–1 h in the dark at 4°C, the cells were washed to remove unspecific staining and transferred to FACS tubes, mixed gently in 400 µl of FACS buffer, and then analyzed by flow cytometry.

### Measurement of ROS levels

To measure ROS production, the cells were incubated with CellROX™ Deep Red reagent (5 µm) for 25 min at 37°C. ROS fluorescence was then detected at 644⁄665 nm of excitation/emission light using a flow cytometer (BD FACSCanto II), and the values were reported as median fluorescence intensity (MFI) and percent of positive cells.

### Determination of intracellular calcium

Fluo4/AM fluorescence (Biotium, Fremont) was used to measure intracellular calcium. In the presence of esterases, Fluo4/AM ester is intracellularly hydrolyzed into Fluo4. When Fluo4 bound to Ca^2+^, the fluorescence increases and becomes detectable. Control and treated cells were resuspended in a 5 mM CaCl_2_ buffer, before incubation with 5 µM of Fluo4/AM for 30 min at 37°C. The cells were washed with PBS, resuspended in 400 µl of CaCl_2_ buffer, and then analyzed by a BD FACSCanto II flow cytometer. The fluorescence was detected at 488/530 nm excitation/emission wavelengths.

### Cell cycle analysis

Control or Ang II-treated cells (1 × 10^6^/ml) were washed with PBS, fixed in ethanol (70%) for 24 h at −20°C. After that, the cells were centrifuged for 5 mins at 400 g, and then washed two times with PBS to remove ethanol and resuspended in 400 µl PBS supplemented with 50 µg/ml of PI. To avoid false RNA staining, the cells were further incubated for 15 min at room temperature in the presence of RNase A (100 µg/ml). Flow cytometry at low speed was performed to analyze the distribution of cells in G1, G2, and S phase of the cell cycle.

### Immunofluorescence imaging

To determine the localization of intracellular calcium, THP1-derived macrophages with and without Ang II treatment were stained by Fluo4/AM. The immunofluorescence imaging was performed using EVOS® system as previously described with modifications (8).

### Fluorescence recovery after photobleaching technique

The experiment was performed as previously described (11). Briefly, monocytes with and without Ang II treatment were stained with 1,1′-dihexadecyl-3,3,3′,3′-tetramethylindocarbocyanine perchlorate (Dil) (2 µM)). After 2 min, the excess of Dil was washed four times. The images were analyzed using the confocal laser scanning microscope (Zeiss) with excitation at 543 nm for Dil and 488 nm for photobleaching, and the emission was collected at 600–700 nm. The PMT voltage was adjusted on the detectors to detect the high background signal with zero measurement following bleaching.

### Adhesion essay

Human umbilical vein endothelial cell (HUVEC) cells were cultured in Dulbecco's Modified Eagle Medium (DMEM) supplemented with 10% fetal bovine serum and 1% pen-strep until they created a monolayer on the bottom of the 6-well plate. The THP-1 cells were cultured in RPMI supplemented with 10% fetal bovine serum and 1% pen-strep; the cells were treated with 100 nM Ang II and/or 100 µM angiotensin receptor blocker (Losartan) and incubated in a humidified chamber at 37°C for 48 h. For labeling, the treated THP1 cells were stained with lipophilic fluorescent stains (DIL), and the cells were then washed and resuspended in complete RPMI and seeded on the attached HUVEC cells in a 6-well plate. The 6-well plate containing attached HUVEC cells and stained THP1 cells was incubated for 30 min at 37°C on shaker at 25 rpm; the plate was then washed three times with 1× phosphate-buffered saline. The images of adherent monocytes were acquired using the EVOS microscope. This experiment was repeated at least three times.

### Human phospho-kinase array

To measure the relative protein phosphorylation, the human phospho-kinase array kit (ARY003C; R&D Systems) was used according to the instruction of the manufacturer (12). Briefly, THP1-derived macrophages treated or not with Ang II (1 μM) were harvested and equal cell lysate (30 μg) from each group was used, than signal intensity was analyzed using the Image Lab software.

### Statistical analysis

The Prism® 5.0 (GraphPad Prism Software Inc., La Jolla, CA, USA) was used for the statistical data analysis. The data are presented as means ± standard deviation (SD). The one-way analysis of variance (ANOVA) was used to compare more than two groups, and the Student *t*-test was used to determine the significance between two groups.

## Results

### Ang II change THP-1-like macrophage phenotype

**Figure 1:**
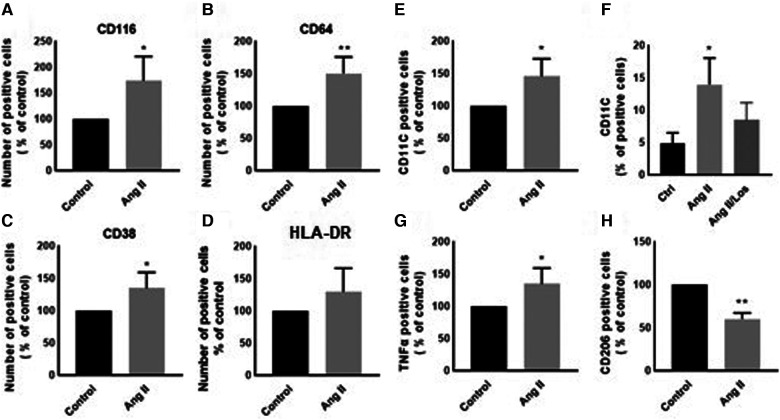
Ang II change THP-1-like macrophage phenotype. The number of positive cells in frequency (%) of (**A**) CD116, (**B**) CD64, (**C**) CD38, (**D**) HLA-DR, (**E,F**) CD11c, (**G**) TNF-α, and (**H**) CD206 were analyzed by flow cytometry using THP-1-derived macrophage treated with angiotensin II (Ang II) (1 µM) for 24 h or untreated cells (Control). In a separate experiment, and before the Ang II treatment, the cells were incubated with Losartan (100 µM) for 2 h (Ang II/Los) (**F**). The results are presented as means ± SEM, *n* = 3–4. *P < 0.05 and **P < 0.01 and compared with controls.

In our study, Ang II increased the expression of CD116 and Fc-gamma receptor I (CD64) (Figures 1A,B), a specific macrophage marker that differentiates it from dendritic cells. Moreover, THP-1-like macrophage cells were highly activated after the treatment with Ang II by an increase in the CD38 marker (Figure 1C). Furthermore, we observed an upregulation of the CD44 marker following Ang II stimulation, which may play a role in proinflammatory phenotype. It has been reported that monocytes differentiated into macrophages that are CD33-deficient increase phagocytosis function and proinflammatory signatures (13). The treatment with Ang II upregulates CD15, CD33, and integrin CD49f (Figures 5A–C), which are known to be involved in monocyte/macrophage differentiation and cell adhesion. The results were confirmed by an adhesion assay showing an increase in monocyte adhesion to endothelial cells after treatment with Ang II and inhibition of this effect by Los treatment (Figures 5E,F). Most of these results were confirmed in human-derived macrophages (Supplementary Figure S2). We therefore hypothesized that Ang II promotes M1-like-THP-1-macrophage phenotype, while suppressing M2 markers. Consistent with this, we observed an upregulation of CD11c, TNF-α, and HLA-DR (HLA class II) (Figures 1D,E,G) as M1 markers and a decrease in the mannose receptor (CD206) (Figure 1H) as an M2 marker. The treatment with angiotensin II type 1 receptor (AT1R) blocker (Losartan) blunted CD11c upregulation (Figure 1F) without any effect on the other markers. The results were confirmed in human-derived macrophages; however, the effect was significantly inhibited by Losartan treatment for both CD11c and TNF-α expression (Supplementary Figure S1).

### Ang II induces apoptosis and cell cycle arrest

**Figure 2:**
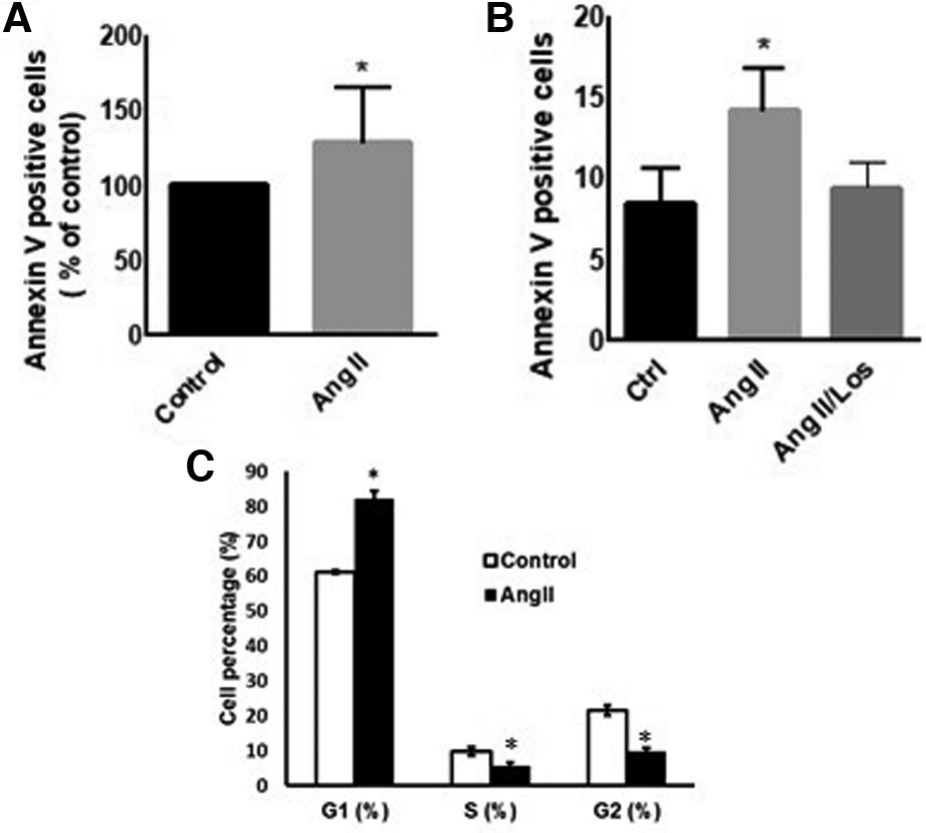
Ang II induces apoptosis and cell cycle arrest. THP-1 macrophages were treated with angiotensin II (Ang II) (1 µM) for 24 h or untreated cells (Control). Apoptosis was measured using Annexin V staining. (**A**) Annexin V positive cells after exposure to Ang II. (**B**) Separate experiment for apoptotic level in cells treated with Ang II (1 µM) for 24 h or Losartan (100 µM) and Ang II (Ang II/Los). (**C**) Cell cycle analysis showing G1, S, and G2 phases. All samples were analyzed using flow cytometry (BD LSR Fortessa). The results are presented as means ± SEM, *n* = 3–4. *P < 0.05 and **P < 0.01 and compared with controls.

To evaluate Ang II effect on cell viability and apoptosis, we treated THP-1 macrophages with 1 µM Ang II for 24 h, 48 h, and 72 h. Apoptosis, measured by Annexin V staining, was significantly detected only after 48 h following treatment with Ang II (Figure 2A). After 24 h of exposure to Ang II, the cell cycle analysis showed G1 arrest in a significant part of macrophages compared with controls (Figure 2C). The apoptosis status was restored after blocking AT1R. The same results of apoptosis were confirmed in human-derived macrophages (Supplementary Figure S5).

### Ang II induced ROS production

**Figure 3:**
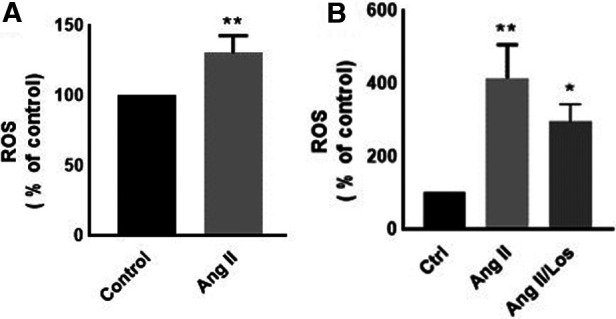
Ang-II-induced ROS production. THP-1 macrophages were treated with angiotensin II (Ang II) (1 µM) for 24 h; Ang II and Losartan (Ang II/Los) (100 µM) or untreated cells (Control). Confluent cells prepared in suspensions at 10^5^–10^6^ cells/ml were incubated with CellROX™ Deep Red reagent (5 μm) for 30 min at 37°C, 5% CO_2_, protected from light, then analyzed using flow cytometry (BD LSR Fortessa). (**A**) ROS production (% of control) in cells treated with Ang II and (**B**) ROS production in a separate experiment for cells treated with Losartan and Ang II. The results are presented as means ± SEM, *n* = 3–4. *P < 0.05 and **P < 0.01 and compared with controls.

Reactive oxygen species (ROS) production is a hallmark of macrophage activation (14). During cellular metabolism, ROS modulates inflammatory macrophage phenotype (15). To evaluate the effect of Ang II on macrophage cellular redox balance in our study, we used the CellROX™ Deep Red for ROS generation analysis. We found that Ang II treatment significantly increased ROS production (Figure 3A). The same results were confirmed in human-derived macrophages (Supplementary Figure S3).

### Ang II increases intracellular calcium and fluorescence recovery after photobleaching

**Figure 4:**
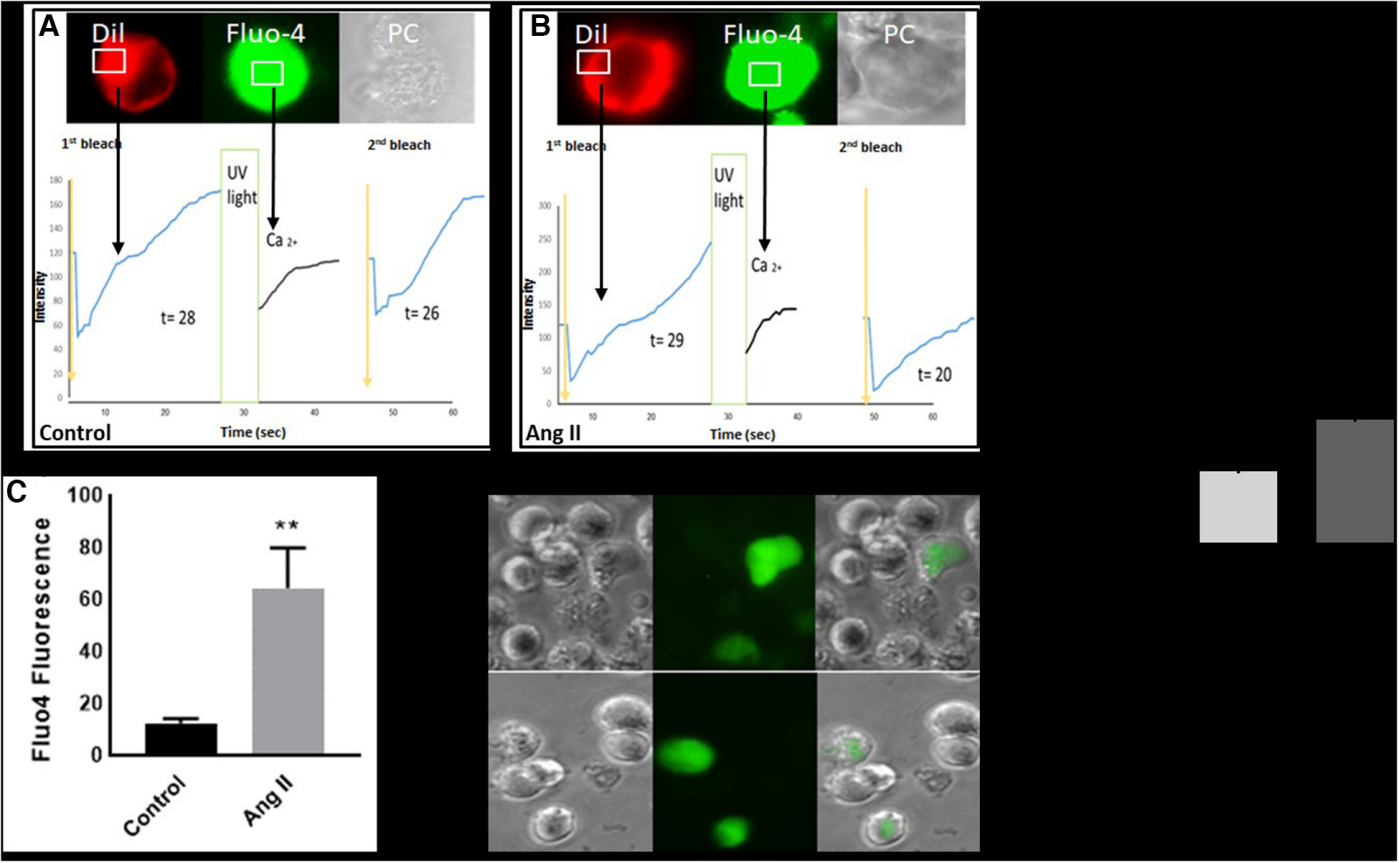
Ang II increases intracellular calcium and fluorescence recovery after photobleaching (FRAP). Cell surface topography during cell elevation of cytosolic Ca^2+^ and change of FRAP signal. The images show fluo4 staining for intracellular calcium, Dil image (Dil) for peripheral cell location, and the phase contrast image (PC). (**A**) Cell surface topography for THP-1 macrophages untreated cells (Control) and (**B**) cells treated with angiotensin II (Ang II) (1 µM) for 24 h. The cells were loaded with the Fluo4 Ca^2+^ indicator, and Dil. After the cell was loaded with calcium indicator Flou4/AM, the cells were tested for FRAP intensity before exposure to UV light (first bleach) and after changing (second bleach). Exposure to UV light by using transient illumination with 404 nm laser. The graphs present the Fluo4 intensity and the time courses of Dil fluorescence. (**D**) and (**E**) Fluo4 fluorescence corresponding to cytosolic free Ca^2+^ measured as median fluorescent intensity. The results are presented as means ± SEM, *n* = 3. *P < 0.05 and **P < 0.01 and compared with controls.

Intracellular calcium modulates the transition of macrophages from a resting state into an activated phase (16). Our results showed that treatment of THP-1-derived macrophages with Ang II increased cytosolic calcium production (Figure 4C). The same experiment was performed and confirmed in human-derived macrophages (Supplementary Figure S4). In addition, the fluorescence recovery after photobleaching (FRAP) technique revealed a delay in calcium expression after UV stimulation (Figures 4A,B). After inducing a Ca^2+^ signal in macrophages, it was noted that the recovery of FRAP signals was faster in the cells treated with Ang II (Figure 4A) compared with control cells (Figure 4B). The full recovery time for treated cells was 20 s, whereas control cells typically completed FRAP recovery no more than 26 s. In a separate experiment, treatment of cells with Ang II induces a decrease in intracellular calcium, partially restored after pretreatment of cells with Los (Figure 4E).

### Ang II increases monocyte adhesion to HUVEC cells

**Figure 5:**
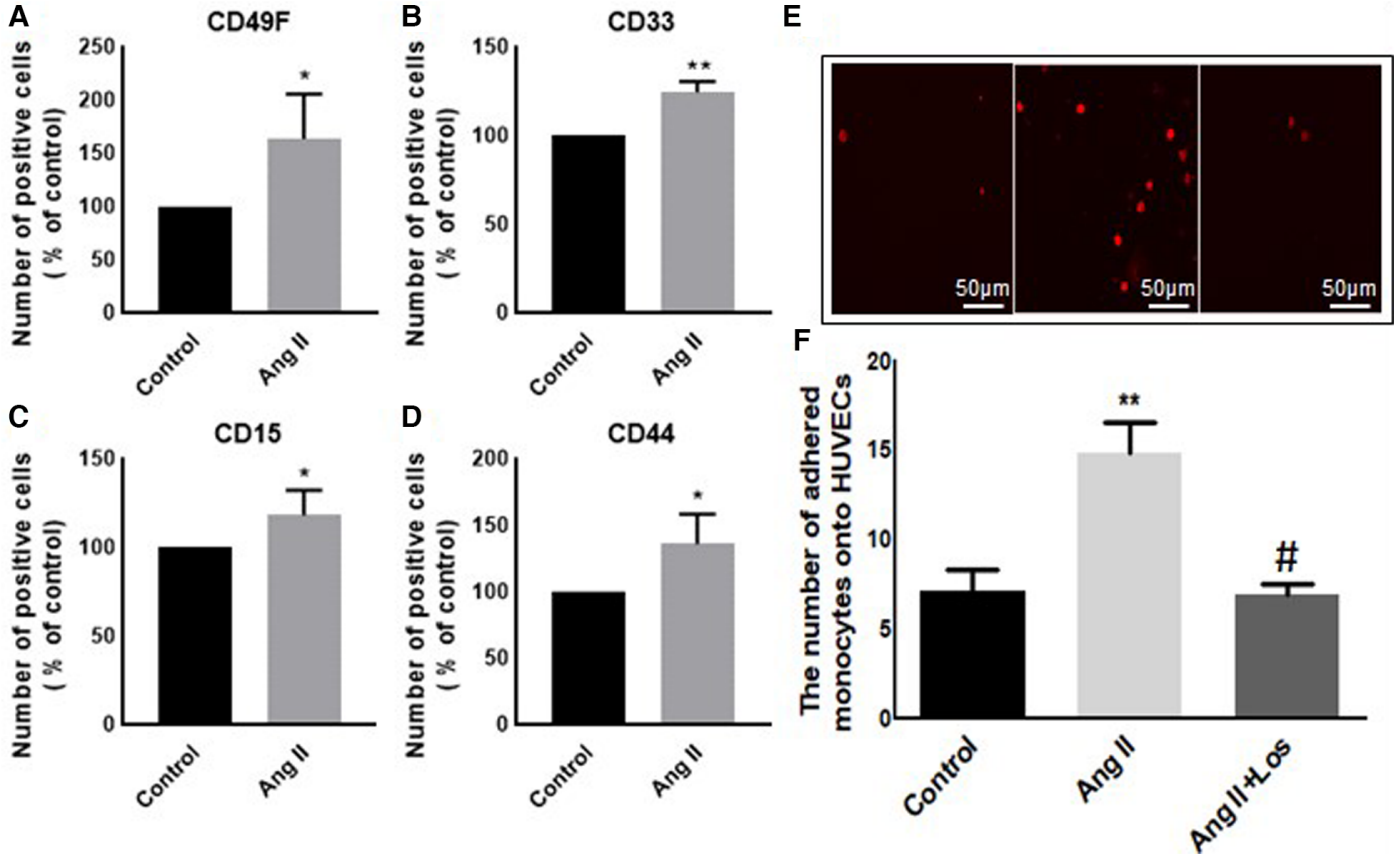
Ang II increases monocyte adhesion to HUVEC cells. The number of positive cells in frequency (%) of (**A**) CD49F, (**B**) CD33, (**C**) CD15, and (**D**) CD44 were analyzed by flow cytometry using THP-1-derived macrophage cells treated with Ang II (1 µM) for 24 h; Losartan (100 µM) and Ang II (Ang II/Los) or untreated cells (Control). (**E,F**) The number of adherent monocytes onto HUVECs, determined using EVOS microscope. The results are presented as means ± SEM, *n* = 4. *P < 0.05 and **P < 0.01 and compared with controls.

Preincubation of monocytes with Ang II induced an increase in binding to endothelial cells (Figures 5E,F). Adhesion of monocyte to the endothelium is a signal of major change in cell phenotype and a first step in initiating inflammation. Treatment with Losartan blunted this effect compared with the Ang II group (Figures 5E,F).

### Ang II increases the phosphorylation level of kinases in THP-1-macrophages

**Figure 6:**
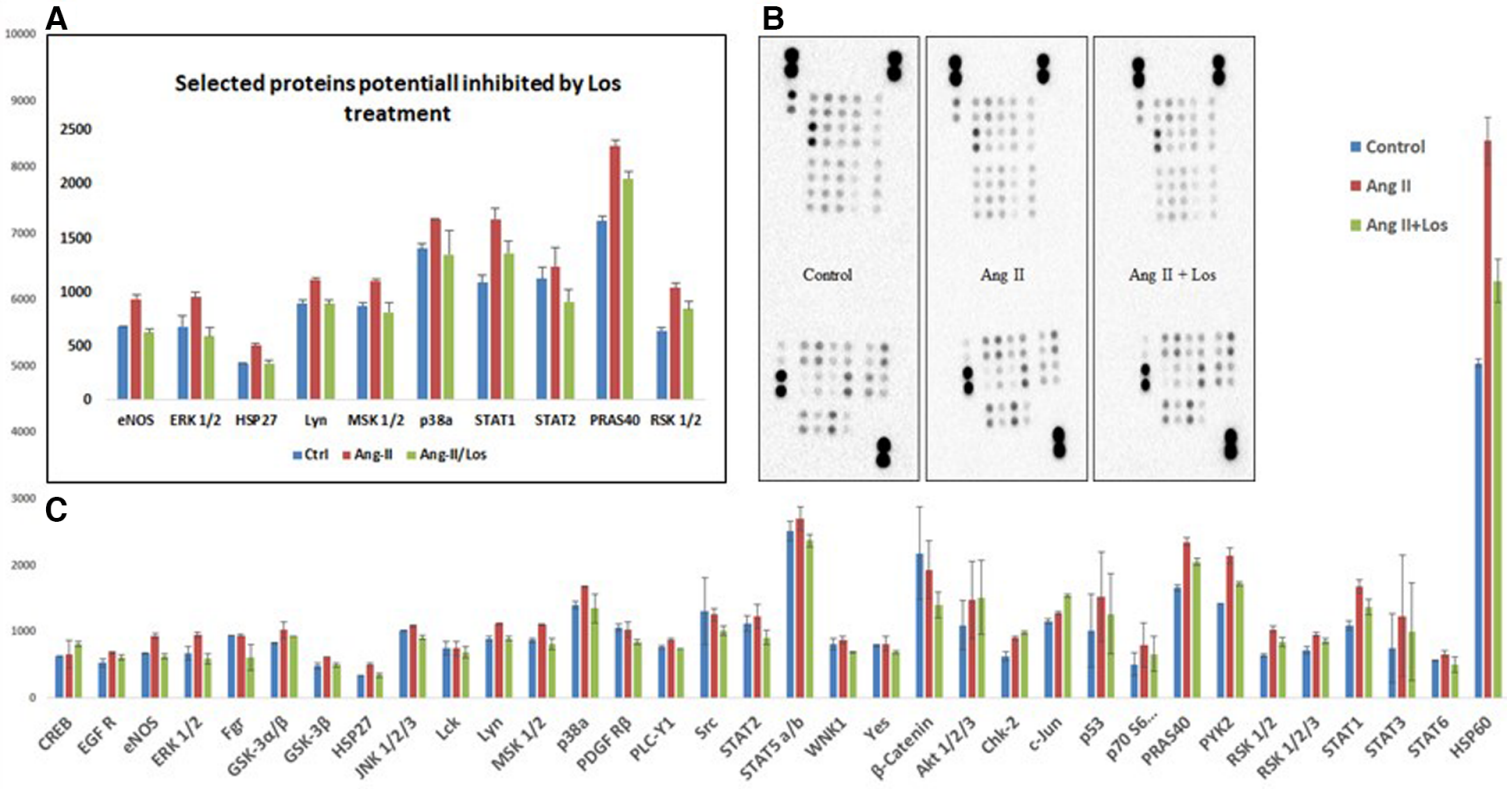
Ang II increases the phosphorylation level of kinases in THP-1 macrophages. Analysis of a proteome profiler for phospho-kinases using THP-1-derived macrophage cells treated with Ang II (1 µM) for 24 h; Losartan (100 µM) for 2 h before the Ang II treatment (Ang II/Los) or untreated cells (Control). Phosphorylated signaling proteins presented as densitometric analysis. (**A**) Selected proteins inhibited by Losartan. (**B**) Data of each array (Part A and B) incubated with 100 μg of cell lysate, shown from a 5-min exposure. (**C**) Proteins panel differentially expressed following the Ang II treatment without significant effect of Losartan. The results are presented as means ± SEM, *P < 0.05, and **P < 0.01 and compared with controls.

We used the Human Phospho-Kinase array to screen the activated proteins in response to Ang-II-stimulated THP-1-macrophages and detect the pathways dependent on AT1R activation. This approach revealed a significant activation of the key proteins of different signaling pathways, such as ERK1/2, p38a, endothelial nitric-oxide synthase (eNOS), STAT1, STAT2, MSK1/2, RSK1/2, Lyn, HSP27, HSP60, and PRAS40 (Figures 6A,B). The activation was blunted after Losartan treatment. In addition, a considerable increase occurred in other phosphoproteins (such as AKT1/2/3, GSK-3α/β, Chk-2, RSK 1/2/3, and STAT3) after treatment with Ang II without any effect of AT1R blocker treatment (Figure 6C).

## Discussion

The activation of the local RAS modulates inflammatory signatures in tissues and cells. Innate immune cells, such as monocytes and macrophages, produce RAS components, such as renin (17), ACE (18), angiotensinogen, Ang II, and ATRs (19, 20). We demonstrate that exogenous Ang II changes monocyte/macrophage phenotype by increasing the expression of CD116, CD64, CD15, CD38, CD44, CD49f, and CD33, most of these parameters were confirmed in human-derived macrophages. The activation of neutrophils via GM-CSFR (CD116) play a crucial role during inflammation (21). We observed an increase in the CD116 marker in THP-1/macrophages treated with Ang II. Yet it has been reported that GM-CSF regulates macrophages differentiation and polarization toward an M1-like phenotype (22) by increasing proinflammatory signatures via modulation of MHC-II (23) through GM-CSFR signaling. Furthermore, GM-CSF increases ROS release in macrophages to drive the phagocytosis process (24). In consonance with these studies, we detected an increased abundance of ROS following the Ang II treatment, which may be due to stimulation of the Ang II/AT1R/NADPH oxidase pathway (14). This effect was rescued following the use of an AT1R blocker (Figure 3B). We therefore suggest that ROS may trigger the proinflammatory phenotype of macrophages, supported by M1-like phenotype markers (CD11c, CD64, TNF-α, and HLA-DR) upregulated after the Ang II treatment, as well as a decrease in the mannose receptor (CD206, an M2 marker). All markers were unaffected by Losartan treatment—except CD11c, which was possibly activated via AT1R; this result was confirmed in human-derived macrophages in addition to TNF-α. ROS modulate the presence and activity of cyclins and cyclin-dependent enzymes involved in cell cycle regulation and progress (25). In addition, it has been reported that ROS production mediates cell cycle arrest (26). In the current study, the Ang II treatment arrested a significant proportion of macrophages in the G1 phase compared with the control group—suggesting a disturbance of the cell cycle profile associated, at least in part, to redox homeostasis dysfunction.

In cardiovascular diseases and especially during low-grade inflammation, it is well known that Ang II modulates the infiltration of monocyte/macrophages into different tissues and organs, such as perivascular tissue, the kidneys, and the heart (6). However, the role of Ang II in macrophage phenotype has still not been well studied. Our results show that Ang II was able to activate THP-1-derived macrophages as well as human-derived macrophages through different pathways. This modulates their function—such as upregulation of the CD11c marker, known to be associated with cytokine production and phagocytosis (27, 28), and TNF-α, the master proinflammatory cytokine released by monocyte/macrophage cells. In our study, the Ang II treatment increased TNF-α expression (an M1 marker), suggesting that a decrease in CD206 (an M2 marker) may be due to downstream pathways activated by TNF-α. It was reported recently that TNF-α−/−M1 macrophages are switched to an M2-like phenotype (29, 30). On the other hand, TNF-α production may be triggered by high-level intracellular ROS generation induced by Ang II.

In macrophages, the Fc-γ receptor I (CD64) plays a crucial role in the clearance of immune composites, as well as the recruitment and activation of inflammatory cells during the inflammatory process (31, 32). This marker was highly expressed following the treatment with Ang II. In addition, activation of the Fc-*γ* receptors leads to stimulation of downstream cell signaling, particularly the NADPH/ROS and calcium signaling pathways (33, 34). In our study, intracellular calcium dysregulation induced by the Ang II treatment was potentially involved in CD64 and CD11c protein expression and ROS production. Similarly, we detected an upregulation of activation and adhesion molecule markers (CD49f, CD44, CD33, and CD38) after the Ang II treatment. We suggest that these markers participate, at least in part, in Ang-II-induced monocyte adhesion to endothelial cells also derived by AT1R. The CD38, robustly expressed in macrophages during inflammation (35), modulates macrophage polarization to an M1-like phenotype, and its overexpression affect proliferation (36)—which may explain, in part, the defect of cell cycle distribution following the Ang II treatment. The CD44 plays a crucial role in THP-1-macrophage differentiation (21), and overexpression of this marker after the Ang II treatment may explain proinflammatory signatures, as the CD44 receptor is involved in the activation of macrophages via the NF-KB pathway (37). Similarly, our results indicate that Ang-II-induced upregulation of CD33, CD49f, and other markers is implicated in monocyte/macrophage differentiation, function, and proinflammatory signatures (13, 38).

**Figure 7:**
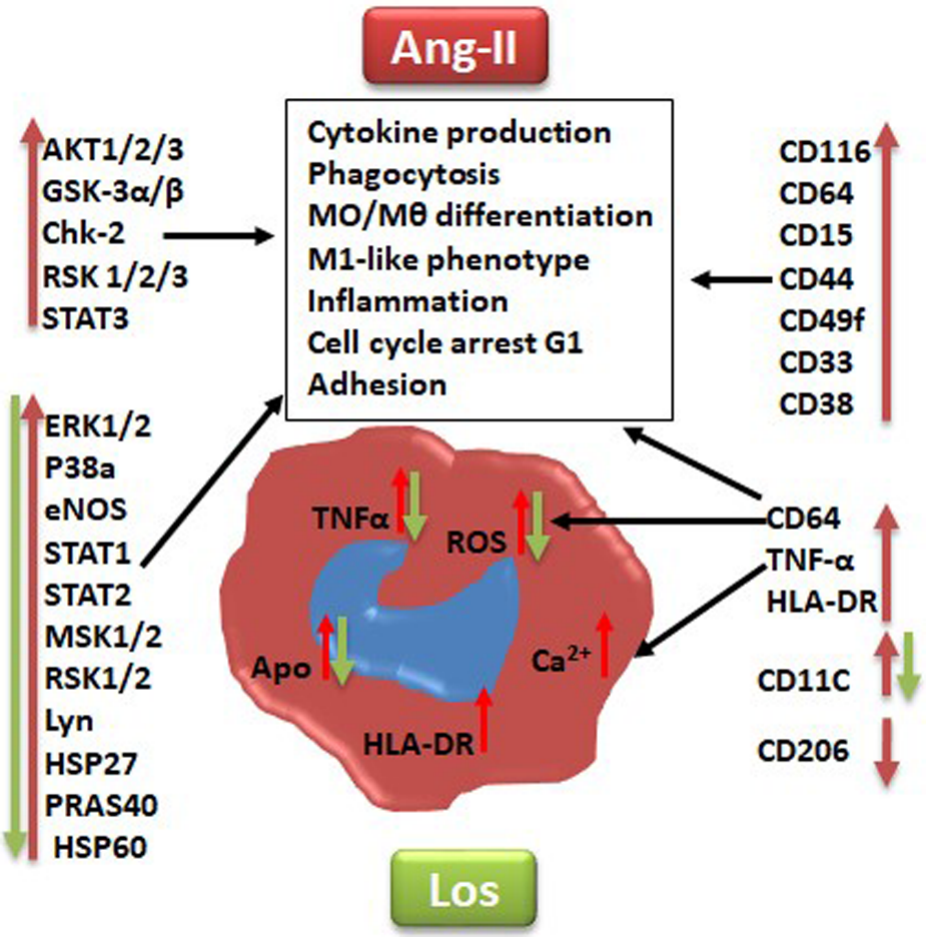
Graphical summary suggested pathways that might be implicated at least in part in angiotensin II inducing monocyte/macrophage dysfunction and polarization (red arrow) and angiotensin type 1 receptor involvement in this process (green arrow). Los, Losartan; ROS, reactive oxygen species; Ca^2+^, intracellular calcium. CD markers for cell phenotype and macrophage polarization: CD116, CD64, CD38, HLA-DR, CD15, CD33, CD49F, CD44, CD11c, TNF-α, and CD206. Human kinases: Akt 1/2/3, GSK-3 alpha/beta, Chk-2 (T68), RSK1/2/3 (S380), ERK1/2 (T202/Y204, T185/Y187), STAT3, p38 alpha, eNOS, STAT1, STAT2, MSK1/2, RSK1/2/3, HSP27 (S78/S82), Lyn (Y397), PRAS40 (T246), and HSP60.

**Figure 8:**
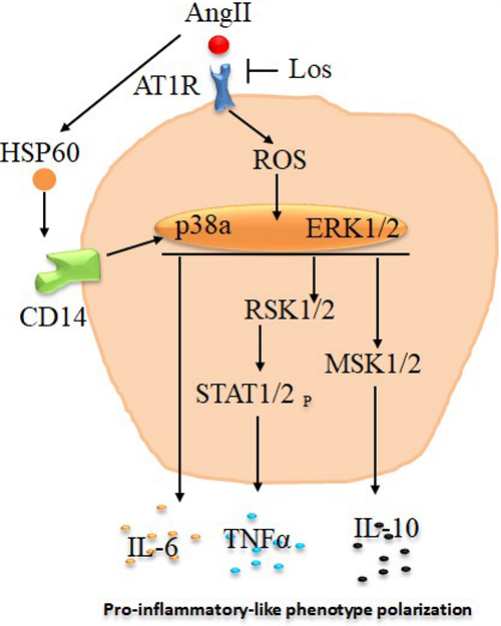
Schematic presentation of potential pathways associated with angiotensin -II-induced macrophages proinflammatory-like phenotype polarization. Angiotensin II (Ang II) stimulate reactive oxygen species (ROS) and heat shock protein 60 (HSP60) via angiotensin II type 1 receptor (AT1R) to stimulate p38a/ERK1/2 pathways, which promote macrophage proinflammatory-like phenotype polarization directly by interleukin 6 (IL-6) release or through RSK1/2 and STAT1/2 phosphorylation to increase tumor necrosis factor alpha (TNF-α) production. These effects are potentially inhibited at least in part by Losartan (Los) treatment.

To deeply investigate signaling pathways and the role of Ang-II-dependent AT1R in monocyte/macrophage differentiation, we performed a phosphoarray analysis. We observed an upregulation of ERK1/2, p38a, eNOS, STAT1, STAT2, MSK1/2, RSK1/2, Lyn, HSP27, HSP60, and PRAS40 (Figure 7), potentially due to AT1R activation. It has been reported that mitogenic stimulation of monocytes/macrophages induces ERK1/2 activation, leading to proinflammatory function (39), and the ERK1/2 and p38a control function of macrophages and IL-10 and TNF-α cytokine release (40). The p38 pathway has been well identified as a key mediator of proinflammatory cytokines, such as TNF-α (41), and it was upregulated by the Ang II treatment and potentially involved in TNF-α increase. Yet it has also been reported that the human Hsp60 protein activates monocytes/macrophages via the CD14/p38 pathway similarly to LPS function and induces IL-6 production (42). We demonstrate an increase in Hsp60 and p38 signaling, which may potentially trigger the proinflammatory profile of the cells upstream stimulated by AT1R (Figure 8). On the other hand, Ang-II-induced overexpression of macrophage eNOS might play a crucial role in the stimulation of proinflammatory protein expression observed following the Ang II treatment. As previously reported, eNOS autoregulated the inflammatory mediators in a model of bone-marrow-derived macrophages (43). In our study, we also show an increase of STAT1 as a key regulator of monocyte differentiation (44), and STAT2 has been associated with dysregulation of macrophage phenotype during viral or bacterial infection (45). RSK1, upregulated by Ang II, is also considered a stimulator of STAT1 phosphorylation, driving proinflammatory activation of macrophages. We suggest that activation of MSK1/2 by the Ang II treatment in our study is due to activation of the ERK1/2 and P38a pathways (Figure 8). The MSK1/2 are mostly involved in counterbalancing the proinflammatory cytokine release, as it has been demonstrated that MSK1/2 regulate IL-10 production in macrophages (46). In addition, in our study, there was an increase in AKT1/2/3, GSK-3α/β, Chk-2, RSK 1/2/3, and STAT3 protein expression after the Ang II treatment; however, these effects are unrelated to AT1R activation and most probably due to AT2R stimulation by Ang II. This result needs further investigation to be validated.

In conclusion, the present study uncovers a key regulator role of Ang II in monocyte/macrophage differentiation and phenotype signatures. These results support the previous studies on the role of Ang II in macrophage functions through AT1R and present a potential approach for targeting other signaling pathways, as we have revealed that not all Ang II effects are dependent on AT1R. *In vivo* studies are advantageous to confirm our finding and to unmask abnormal immune responses in monocytes/macrophages.

## Data Availability

The original contributions presented in the study are included in the article/**Supplementary Material**, further inquiries can be directed to the corresponding author.
