# Supplementary material for: Angiotensin II modulates THP-1-like macrophage phenotype and inflammatory signatures via angiotensin II type 1 receptor
Source: Front Cardiovasc Med. 2023 Aug 25;10:1129704. doi: 10.3389/fcvm.2023.1129704 (PMC10485254; doi:10.3389/fcvm.2023.1129704)
Supplement: Supplementary file 1 [file Datasheet1.docx]

**Angiotensin II modulate Thp-1-like macrophage phenotype and inflammatory signatures via angiotensin II type1 receptor (AT1R)**


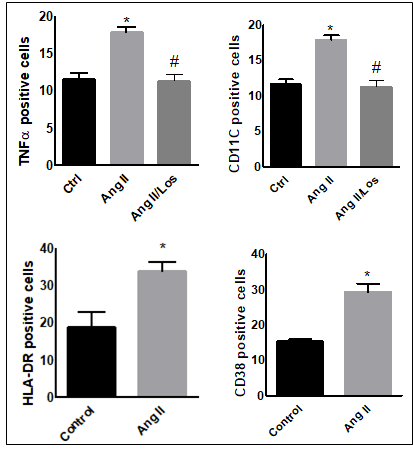


**Supplementary figure 1: Angiotensin II change polarization of human monocyte/macrophages**

The positive cells in frequency of HLA-DR, TNF-α, CD11C and CD38 were analyzed by flow cytometry using human-derived macrophage treated with Angiotensin-II (Ang II) (1µM) for 24h or untreated cells (Control). In separate experiment, and before Ang II treatment, cells were incubated with losartan (100 µM) for 2h (Ang II/Los). Results are presented as means ± SEM, n=3-4. *P < 0.05 compared to controls and ^#^P<0.05 compared to Ang-II.


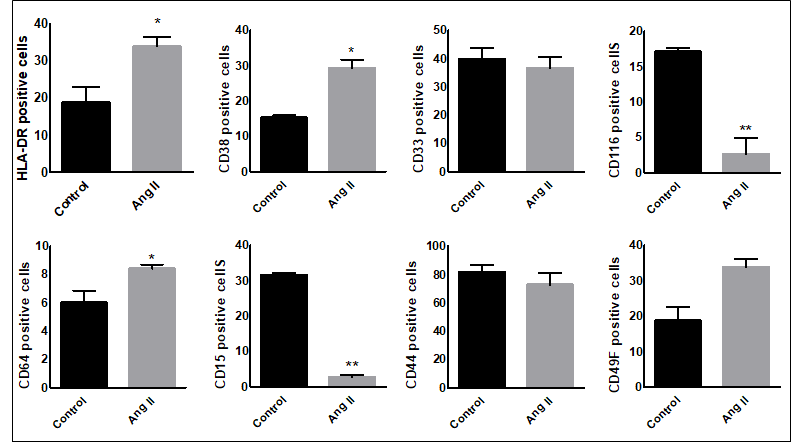


**Supplementary figure 2: Angiotensin II change human monocyte/macrophages phenotype**.

The positive cells in frequency of HLA-DR, CD33, CD116, CD64, CD15, CD44 and CD49F were analyzed by flow cytometry using human-derived macrophage treated with Angiotensin-II (Ang II) (1µM) for 24h or untreated cells (Control). Results are presented as means ± SEM, n=3-4. **P* < 0.05 and ***P* < 0.01 and compared to controls.


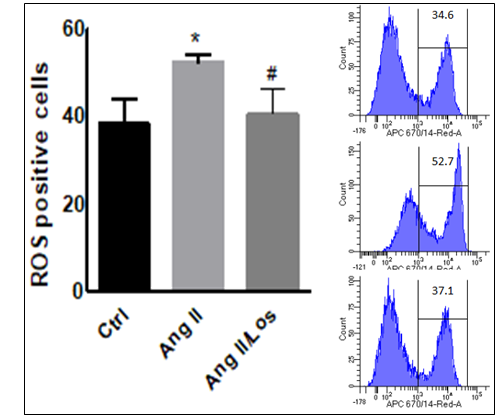


**Supplementary figure 3: Ang II induced ROS production in human monocyte/macrophages.**

Human monocyte/macrophages were treated with Angiotensin II (Ang II) (1µM) for 24h; Ang II and losartan (Ang II/Los) (100 µM) or untreated cells (Control). Cells prepared in suspensions at 10^5^ to 10^6^ cells/mL were incubated with CellROX Deep Red reagent (5 μm) for 30 minutes at 37°C, 5% CO2, protected from light, than analyzed using flow cytometry (BD LSR Fortessa). Results are presented as means ± SEM, n=3-4. *P < 0.05 compared to controls and ^#^P<0.05 compared to Ang-II.


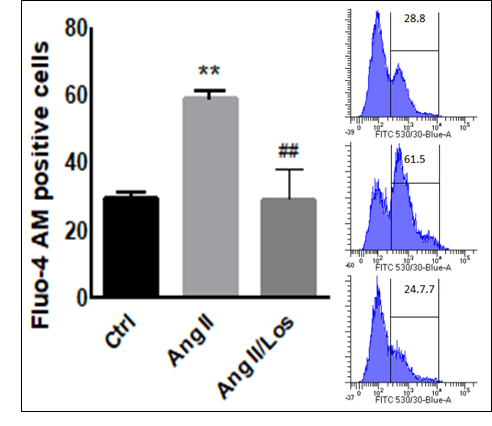


**Supplementary figure 4: Ang II increases intracellular calcium in human monocyte/macrophages**

Human monocyte/macrophages treated with Angiotensin II (Ang II) (1µM) for 24h; Ang II and losartan (Ang II/Los) (100 µM) or untreated cells (Control). Cells were loaded with the Fluo4 Ca2+ indicator, and Dil. Human monocyte/macrophages was loaded with calcium indicator Flou4 AM, Fluo4 fluorescence corresponding to cytosolic free Ca2+ measured as median fluorescent intensity. Results are presented as means ± SEM, n=3-4. **P < 0.01 compared to controls and ^##^P<0.01 compared to Ang-II.


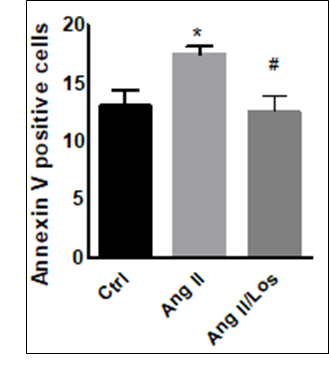


**Supplementary figure 5: Ang II induces Apoptosis in human monocyte/macrophages.**

Human monocyte/macrophages were treated with Angiotensin II (Ang II) (1µM) for 24h; Ang II and losartan (Ang II/Los) (100 µM) or untreated cells (Control). Apoptosis was measured using Annexin V staining. All samples were analysed using flow cytometry (BDLSR Fortessa). Results are presented as means ± SEM, n=3-4. *P < 0.05 compared to controls and ^#^P<0.05 compared to Ang-II.
